# Clinical Results in Medullary Thyroid Carcinoma Suggest High Potential of Pretargeted Immuno-PET for Tumor Imaging and Theranostic Approaches

**DOI:** 10.3389/fmed.2019.00124

**Published:** 2019-06-04

**Authors:** Caroline Bodet-Milin, Clément Bailly, Yann Touchefeu, Eric Frampas, Mickael Bourgeois, Aurore Rauscher, Franck Lacoeuille, Delphine Drui, Nicolas Arlicot, David M. Goldenberg, Alain Faivre-Chauvet, Jacques Barbet, Caroline Rousseau, Françoise Kraeber-Bodéré

**Affiliations:** ^1^Nuclear Medicine, University Hospital, Nantes, France; ^2^CRCINA, INSERM, CNRS, Université d'Angers, Université de Nantes, Nantes, France; ^3^Hepato-Gastro-enterology, University Hospital, Nantes, France; ^4^Radiology, University Hospital, Nantes, France; ^5^Nuclear Medicine, ICO Cancer Center, Saint-Herblain, France; ^6^Nuclear Medicine, University Hospital, Angers, France; ^7^Endocrinology Department, University Hospital, Nantes, France; ^8^Nuclear Medicine, University Hospital, Tours, France; ^9^IBC Pharmaceuticals, Inc., Morris Plains, NJ, United States; ^10^Immunomedics, Inc., Morris Plains, NJ, United States; ^11^GIP Arronax, Saint-Herblain, France

**Keywords:** medullary thyroid carcinoma (MTC), immunoPET, theranostic (therapeutic and diagnostic), pretargeted imaging, radioimmunoconjugate

## Abstract

Monoclonal antibody (mAb)-based therapies have experienced considerable growth in cancer management. When labeled with radionuclides, mAbs also represent promising probes for imaging or theranostic approaches. Initially, mAbs have been radiolabeled with single-photon emitters, such as ^131^I, ^99m^Tc, or ^111^In, for diagnostic purposes or to improve radioimmunotherapy (RIT) using dosimetry estimations. Today, more accurate imaging is achieved using positron- emission tomography (PET). Thanks to the important technical advances in the production of PET emitters and their related radiolabeling methods, the last decade has witnessed the development of a broad range of new probes for specific PET imaging. Immuno-PET, which combines the high sensitivity and resolution of a PET camera with the specificity of a monoclonal antibody, is fully in line with this approach. As RIT, immuno-PET can be performed using directly radiolabeled mAbs or using pretargeting to improve imaging contrast. Pretargeted immuno-PET has been developed against different antigens, and promising results have been reported in tumor expressing carcinoembryonic antigen (CEA; CEACAM5) using a bispecific mAb (BsmAb) and a radiolabeled peptide. Medullary thyroid carcinoma (MTC) is an uncommon thyroid cancer subtype which accounts for <10% of all thyroid neoplasms. Characterized by an intense expression of CEA, MTC represents a relevant tumor model for immuno-PET. High sensitivity of pretargeted immunoscintigraphy using murine or chimeric anti-CEA BsMAb and pretargeted haptens-peptides labeled with ^111^In or ^131^I were reported in metastatic MTC patients 20 years ago. Recently, an innovative clinical study reported high tumor uptake and contrast using pretargeted anti-CEA immuno-PET in relapsed MTC patients. This review focuses on MTC as an example, but the same pretargeting technique has been applied with success for clinical PET imaging of other CEA-expressing tumors and other pretargeting systems. In particular, those exploiting bioorthogonal chemistry also appear interesting in preclinical animal models, suggesting the high potential of pretargeting for diagnostic and theranostic applications.

## Introduction

Targeting radionuclides to tumor cells using monoclonal antibodies (mAbs) has emerged for imaging and therapy purposes (1). The production of chimeric or humanized mAbs with lower immunogenicity than murine mAbs prompted the clinical development of immunotherapy, and the anti-tumor effects reported with trastuzumab in breast cancer (BC) expressing HER2 and of the anti-CD20 rituximab in B-cell non-Hodgkin lymphoma demonstrated for the first time the high potential of mAbs for cancer therapy. The clinical successes of rituximab and trastuzumab have accelerated the research for new target membrane proteins in different types of malignant tumors. Some monoclonal antibodies have also been radiolabeled for tumor imaging by scintigraphy with promising initial results. Yet, in spite of mAbs' good specificity, the expected success was limited by the low resolution of the images. Thanks to significant technical progress in the production of positron emitters and their labeling methods, as well as the development of more sensitive detectors and specific software, the last decade has seen the development of a wide range of new PET radiopharmaceuticals. In medical practice, the identification of biomarkers is gradually becoming a prerequisite for any treatment decision, along with the approach of personalized medicine. Immuno-PET, which combines the high sensitivity and resolution of a PET camera with mAb's specificity, is an excellent candidate for this new concept (2, 3). mAbs labeled with radionuclides represent promising probes for theranostic approaches, providing a non-invasive solution for *in vivo* evaluation of target expression, distribution and accessibility, and for obtaining reliable information for diagnosis, prognosis, and therapy. Based on immunoPET, treatment strategies could be adapted to each patient before pricey and potentially toxic treatments are administered (4, 5).

Medullary thyroid carcinoma (MTC) accounts for <10% of all thyroid cancers (6). After initial surgery, serum calcitonin is used to monitor residual disease, which is still detectable in nearly 20% of patients after surgery. Imaging including neck ultrasound, neck and chest computed tomography (CT), liver contrast-enhanced CT or magnetic resonance imaging (MRI), and spine and pelvis bone MRI are recommended when calcitonin exceeds 150 pg/ml (7). Due to their ability to characterize and quantify cancer molecular processes, ^18^F-DOPA or ^18^F-FDG PET tracers also have a major interest in patients with recurrent MTC and offer great potential as surrogate biomarkers, useful for early response evaluation and prediction of outcomes (8–11).

MTC is characterized by a high and homogeneous expression of ACE. Several clinical trials have shown pretargeted immunoscintigraphy's sensitivity, performed using the Affinity Enhancement System (AES) based on the injection of murine or chimeric anti-CEA bispecific antibodies (BsMAb) and pretargeted haptenpeptides radiolabeled with ^111^In or ^131^I (12, 13). Prolonged tumor efficacy was also observed using therapeutic haptens radiolabeled with ^131^I (14). These results and the high potential of pretargeting reported in other solid tumors using different radioimmunoconjugates suggested that pretargeted peptides labeled with PET emitters would take advantage of the better sensitivity and resolution of PET compared to SPECT and provide high sensitivity and specificity imaging under good conditions of radiation protection and dosimetry (15–17). However, no clinical study has yet compared pretargeted immuno-PET with pretargeted immuno-SPECT.

This review focuses on MTC as an example, but the same pretargeting technique has been applied with success for clinical PET imaging of other CEA-expressing tumors and, in mice, to other target antigens (18). Other pretargeting systems, in particular those exploiting bio-orthogonal chemistry, also appear interesting in preclinical animal models, suggesting the high potential of pretargeting for diagnostic or theranostic applications (19).

### Choice of Radionuclide for Immuno-PET

For nearly 30 years, mAbs have been labeled with gamma-emitting radionuclides, such as ^131^I, ^99m^Tc, or ^111^In for planar or Single Photon Emission Computed Tomography (SPECT) imaging. However, the sensitivity of these techniques is low, the resolution poor and accurate quantitative information cannot be obtained. PET provides quantitative information and has a better spatial resolution that allows for good delineation of tumors and organs. In addition, exact attenuation correction, precise dispersion correction, improved sensitivity, good signal-to-noise ratios, and the ability to perform true whole body imaging within a reasonable time frame are key factors in the outperformance of PET over SPECT.

Marrying mAbs and PET emitters requires an appropriate match between the biologic half-life of the protein and the physical half-life of the isotope to achieve optimal tumor-to-background activity ratios (4, 5). Indeed, intact mAbs have a circulation time of several days, and longer imaging windows allow for both the accumulation of the tracer in the target tissue and the clearance of unbound tracer from the blood pool. This in turn leads to improved image contrast and tumor-to-background activity ratios. ^89^Zr and ^124^I are well suited to the labeling of large molecules, such as intact mAbs. The long half-life also offers an advantage for logistics related to transportation. ^64^Cu, with an intermediate half- life of 12.7 h, can be used for labeling a large number of molecules of different sizes ^18^F or ^68^Ga, with their short half-life, may be used to label small-size molecules, such as peptides or small molecular weight binding proteins, that distribute rapidly in the body. They are appropriate for pretargeted PET imaging, as shown for ^68^Ga in the studies discussed here. ^18^F may also be used to label the haptens or small molecular weight tracers for pretargeting and, for example, this was done in preclinical studies with a NOTA-derivatized hapten by the formation of an aluminum-fluoride complex (20, 21).

From a “theranostic” perspective, the pairs of beta+/beta emitting radionuclides (^124^I/^131^I, ^86^Y/^90^Y, ^64^Cu/^67^Cu, ^44^Sc/^47^ Sc) are very promising as the same distribution is expected both for imaging dosimetry and therapy.

Other considerations must also be taken into account when selecting appropriate radionuclides. In addition to the half-life, the existence of concomitant gamma emissions will have significant effects on the radiation dose received by the patient. Positron range may affect resolution if the positron travels a significant distance before annihilation. Finally, additional factors to consider include cost and availability.

### Pretargeting for Immuno-PET

Since the first clinical pretargeted scintigraphy and radioimmunotherapy clinical studies discussed previously (12–14), new pretargeting reagents for the AES method have been designed (20–23). TF2 is an engineered BsMAb composed of anti-hapten Fab-fragment derived from the murine 679 antibody recognizing the histamine-succinyl-glycine (HSG) motif, and two humanized anti-CEA Fab-fragments derived from the hMN-14 antibody, formed into a trivalent 157 kD protein by the Dock-and-Lock® procedure (22). IMP288 is a bivalent HSG hapten that can be labeled with a variety of radionuclides for therapy (^90^Y and ^177^Lu), scintigraphy (^111^In) or PET (^124^I, ^68^Ga, and ^18^F) (20, 21, 23). The clinical implementation of pretargeting requires a first phase to optimize the BsMAb and peptide molar doses and a delay between the two injections (24–27). The first clinical results were reported using TF2/^177^Lu-IMP288 in colorectal carcinoma patients. Fast tumor uptake and high tumor-to-background activity ratios were observed within a few hours (24, 25). These results using TF2/^177^Lu-IMP288 were confirmed in a phase I clinical trial performed in patients with CEA-positive lung cancer (27). This phase I study determined that a pretargeting delay of 24 h between the TF2 and the radiolabeled peptide injections was considered the best compromise between the high tumor uptake required to deliver a high irradiation dose to tumor cells and a high tumor-to-background activity ratio to reduce irradiation of normal tissues. Along the same line, high doses of TF2 (75 mg/m^2^) were used to deliver sufficient irradiation using a 10:1 TF2: hapten molar ratio. The rapid distribution of the reagents observed in these therapy trials, indicated that that labeling with the short-lived radionuclides, ^68^Ga or ^18^F, for PET should be feasible. Whereas, ^18^F allows PET imaging with better resolution than ^68^Ga possibly with lower cost, the choice between ^68^Ga and ^18^F would mostly depend on the logistics of the clinical centers. With ^68^Ga having the advantage of availability via a generator (20), the imaging performance of immuno-PET using TF2/^68^Ga-IMP288 was tested in an orthotopic murine xenograft model of human colonic liver metastases (28). ^68^Ga-immuno-PET allowed for better tumor/organ ratios compared to ^18^FDG-PET (*P* < 0.05) for both imaging and biodistribution. Sixty-seven percent of tumors were detected with ^68^Ga-immuno-PET vs. 31% with ^18^FDG PET (*P* = 0.049). For tumors <200 mg, the sensitivity was 44% with ^68^Ga-immuno-PET vs. 0% for ^18^FDG PET (*P* = 0.031). Finally, tumor uptake measured on PET images was strongly correlated to biodistribution analyses (*r*^2^ = 0.85).

### Preliminary Results of Pretargeting Immuno-PET in MTC Patients

**Figure 1:**
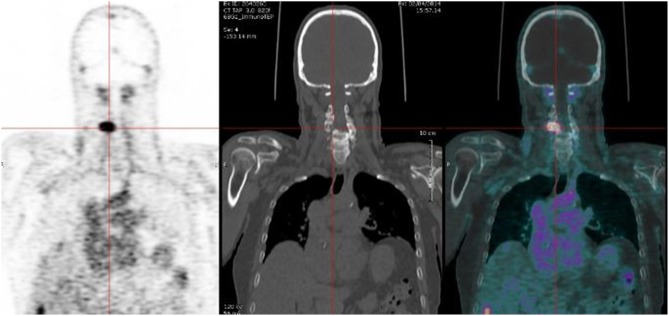
Immuno-PET performed using TF2 BsMAb and ^68^Ga-IMP288 peptide in a MTC patient with a spinal lesion.

A pilot clinical study was designed in relapsed MTC patients to transfer TF2 /^68^Ga-IMP288 pretargeting to the clinic. The first part of the study aimed at determining the best pretargeting parameters. Different cohorts of patients were injected with variable TF2 and IMP288 molar doses at variable pretargeting delays. The second part was designed to assess immuno-PET performance. Adults with a histological diagnosis of MTC treated by complete surgery and presenting a calcitonin serum level ≥150 pg/ml, with at least one lesion ≥10 mm on conventional imaging, were eligible. The results of the first part of the study have been published, and the analysis of the second part is in progress (29). First, the molar doses of TF2 and hapten were reduced as compared to the therapy studies, because the injected activity of short-lived ^68^Ga was set to 150 MBq, as compared to GBq activities of ^177^Lu for therapy. According to a PET semi-quantitative analysis and pharmacokinetic studies, the 30-h pretargeting delay between BsMAb and peptide injections was the most favorable for imaging: tumor uptake was not significantly reduced as compared to 24-h and tumor/background ratios were better. Pretargeted immuno-PET detected MTC confirmed foci in all patients except one. Our previous studies showed that CEA expression seemed to be almost constant in MTC, and that high sensitivity PET imaging using CEA as a target would detect the disease independently of the prognosis, in contrast to ^18^FDG or ^18^F-DOPA PET/CT (10, 12, 13, 29). The preliminary results obtained in the first 12 MTC patients already suggested that high tumor contrast can be obtained using this novel whole-body imaging (Figure 1) (29, 30). In this small cohort of metastatic patients with a median calcitonin of 915 pg/ml (249–5,300) and CEA of 29.5 ng/ml (7.4–257), a total of 110 lesions were detected by immuno-PET, whereas CT detected 59 lesions, bone MRI 12 lesions, liver MRI 13 lesions, and ^18^F-DOPA-PET/CT 63 lesions. Since pathological confirmation was generally not possible, in the MTC studies and in other pathologies such as colorectal cancer and breast cancer discussed below, the Gold Standard was defined as follow. A lesion detected by immuno-PET was considered to be related to cancer when it was confirmed by histology and/or detected by another imaging method and/or confirmed by follow-up. Complementary imaging (for example CT, MRI, ^18^F-DOPA PET and ^18^FDG PET in the MTC study) could be prescribed within 3 months after immuno-PET to confirm anomalies detected by immuno-PET but not visualized on the inclusion imaging assessment. The preliminary analysis then resulted in an overall sensitivity of 89% for immuno-PET, with 100% sensitivity for lymph nodes and liver, 87% for bone, and 42% for lungs. Overall sensitivities of CT, bone MRI, liver MRI and ^18^F-DOPA-PET/CT were 77, 92, 76, and 66%, respectively.

### Promising Performance of Pretargeted ^68^Ga-IMP288 in Other CEA-Positive Tumors

**Figure 2:**
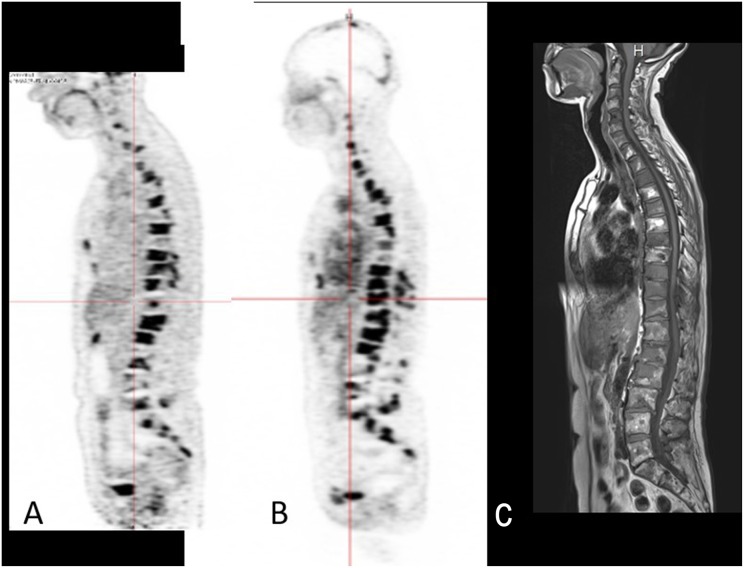
**(A)** FDG PET/CT, **(B)** Immuno-PET with TF2 and ^68^Ga-IMP288 peptide showing multiple spine bone foci, and **(C)** STIR TSE bone MRI showing multiple spine bone abnormalities in a BC patient.

CEA is expressed in other solid tumors, and pretargeted immuno-PET has also been assessed with promising results in BC and colorectal carcinoma (CRC). Preliminary results have been reported at international congresses (31, 32). In the 9 metastatic BC patients enrolled in an optimization immuno-PET study evaluating TF2 /^68^Ga-IMP288, with median CA15-3 was 249.3 kUI/L (40–2,448) and a median CEA of 76 μg/L (9.5–1359.0), pretargeted anti-CEA immuno-PET allowed the detection of a total of 533 lesions, whereas 245 lesions were detected by CT, 160 by bone MRI, and 425 by ^**18**^FDG-PET/CT (Figure 2). Immuno-PET showed 92.5% overall sensitivity, with, respectively, 100% sensitivity for bone, liver, skin, and brain, 91% for lymph nodes, and 28.5% for lung. Brain lesions were only seen on immuno-PET imaging and secondly confirmed by MRI.

Another pilot study included 11 patients who received an imaging work-up for the diagnosis of metastases from CEA-expressing CRC, comprising TF2/^68^Ga-IMP288 immuno-PET, ^18^FDG-PET, thoraco-abdominopelvic CT, liver MRI, and abdominal ultrasound scanning. In the per-patient analysis, immuno-PET was positive in 9/11 patients. The two negative patients were, respectively, one false-negative (single lung metastasis) and one true-negative (mediastinal sarcoidosis). On a per-lesion analysis, the sensitivity, specificity, positive predictive value and negative predictive value were, respectively, 82, 25, 82, and 25% for the morphological assessment (CT + ultrasound + MRI); 76, 67, 87, and 33% for ^18^FDG-PET; and 88, 100, 100, 67% for immuno-PET (32).

These data show high performances of pretargeted immuno-PET in tumor detection, except for lung lesions. Several hypotheses can explain the low sensitivity in this organ. On the one hand, the current generations of CT scanners are very sensitive and can diagnose very small pulmonary nodules. Thus, since our Gold standard does not require histological evidence, micronodules considered as discretely progressive on follow up CT scans were validated as related to tumor. The very small size of some of these lesions could explain the low sensitivity of immuno-PET since immuno-PET images were recorded in “spontaneous breathing” thus underestimating the uptake in infra-centimetric pulmonary nodules due to the partial volume effect related to the amplitude of respiratory movements. On the other hand, immunoscintigraphy images recorded 5–10 days after hapten injection showed better sensitivity in lung lesions (10). So, it is possible that immuno-PET images have been recorded too early after hapten injection to allow the visualization of some infra-centimetric pulmonary nodules. Later images may have improved tumor contrast. A longer half-life radionuclide like ^64^Cu, permitting later images, could improve PET sensitivity to detect lung nodules.

### Other Pretargeting Systems

Pretargeted immuno-PET using the AES method also has been successfully assessed against other tumor antigens in preclinical models (18). TF12 is a trivalent BsMAb consisting in two anti-TROP-2 Fab fragments and one anti-HSG Fab fragment. Many epithelial cancers, including prostate cancer (PC) express the TROP-2 antigen. The potential of pretargeted immuno-PET with TF2 /^68^Ga-IMP288, was studied in mice with subcutaneous and intraperitoneal PC3 human prostate tumors, using ^18^FDG-PET as a reference. ^68^Ga-IMP288 demonstrated a rapid accumulation in the TF12 pretargeted subcutaneous tumors (7.2 ± 1.1% ID/g), and low blood levels and kidney uptake resulting in high tumor/blood ratios (17.4 ± 11.2) at 1 h p.i. ^18^FDG's uptake was significantly lower (3.4 ± 0.9% ID/g, *P* = 0.008), with lower tumor/blood ratios (3.0 ± 1.9, *P* = 0.011). Immuno-PET identified both subcutaneous and intraperitoneal tumors as small as 5 mm^3^, suggesting that the method was efficient for rapid, sensitive, and specific imaging of PC.

Recently, entirely different pretargeting approaches have been developed or revisited. One is based on the *in vivo* formation of an oligonucleotide duplex. A first oligonucleotide analog (e.g., peptide nucleic acid or PNA) is coupled to an antibody or binding protein (an anti-HER2 Affibody) for pretargeting of a radiolabeled complementary oligonucleotide analog (33). Good tumor targeting was achieved with a significant reduction in blood and kidney retention 1 h after activity injection, as compared to the directly-labeled Affibody in a human ovarian cancer model in mice. The other approach, which attracts even more interest, is based on bio-orthogonal chemistry, also known as click chemistry. While click chemistry became quite popular about 15 years ago for various coupling reactions *in vitro*, it was soon discovered that these very fast chemical reactions can occur *in vivo* as well with similar efficiency and specificity. They were soon proposed for pretargeting applications (34). The CC49 antibody recognizing the TAG72 antigen derivatized with trans-cyclooctene (TCO) was used for pretargeting ^111^In-labeled DOTA-dipyridyltetrazine, demonstrating fast and high tumor activity uptake and high tumor-to-muscle ratios in a mouse model. This pioneering work was followed by a large number of preclinical investigations aiming at further improving the pretargeting performance by testing alternative bio-orthogonal chemistry reagents, adding a chase step between the injection of the antibody and that of the labeled compound, and also by applying bio-orthogonal pretargeting to small-binding proteins, such as diabodies or Affibodies. These efforts have been reviewed recently in a broad comparison of all pretargeting approaches by Altai and coworkers (19). Translation of such new pretargeting approaches to the clinic should come soon, both for PET imaging and therapy.

## Conclusion

An increased interest for immuno-PET is found in the recent literature (17), where targeted therapies using antibodies are experiencing a considerable growth in cancer management. Immuno-PET can offer a non-invasive solution to quantitatively assess whole-body tumor biomarker cartography. Based on immuno-PET, treatment strategies could be adapted to each patient before costly and potentially toxic treatments are administered.

Pretargeted immuno-PET represents a sensitive and specific imaging method, with promising results reported in MTC and also in other solid tumors. Pretargeted immuno-PET could indeed be a specific diagnostic tool for tumor detection, but also a theranostic companion approach to select patients to be treated with radioimmunconjugates or antibody-drug conjugates.

Pretargeting has advantages and limitations. It can be used to visualize or treat tumor lesions, depending on the radionuclide used, for example by using ^111^In and ^99m^Tc for SPECT imaging, ^68^Ga or ^18^F for PET imaging, or ^131^I, ^90^Y, and ^177^Lu for radioimmunotherapy. Using BsMab as the pretargeting agent has several advantages: BsMAb can be humanized to minimize their immunogenicity and tailored to clear from the circulation more rapidly than intact IgG's. Then, together with the limited affinity of the BsMAb-hapten binding that allows for dissociation of BsMAb -hapten complexes in the circulation, there is no need for a clearing agent. Over the past decade, several improvements have been made to this system, resulting in a flexible, and efficient pretargeting system. However, it requires careful optimization, both for the design of the appropriate pretargeting reagents and for the definition of dosing and administration schedules. In addition, optimal reagents doses and injection schedules are not identical for imaging, where rapidly achieving high tumor to non-tumor activity ratios is the goal, and therapy where sufficient irradiation of tumors is also needed.Several roads of improvement exist. The non-covalent binding between the radiolabeled hapten and BsMAb on the surface of tumor cells limits the retention of the radiolabeled hapten-peptide in the tumor. Recent developments in the use of bio-orthogonal chemistry are very promising and represent an attractive alternative to the use of BsMAb. The question of cost should also be examined. Although these innovative technologies are certainly costly, but this could be acceptable if the advantage in patient selection for expensive therapies and drug development is confirmed. Large-scale, randomized, multicenter clinical trials are warranted.

## Author Contributions

All authors listed have made a substantial, direct and intellectual contribution to the work, and approved it for publication.

### Conflict of Interest Statement

At the time the work was conducted, DG was Chairman of the Board, Chief Scientific Officer, and Chief Patent Officer of Immunomedics, Inc., and also Chairman of IBC Pharmaceuticals, Inc. The remaining authors declare that the research was conducted in the absence of any commercial or financial relationships that could be construed as a potential conflict of interest.
